# Full-state time-varying asymmetric constraint control for non-strict feedback nonlinear systems based on dynamic surface method

**DOI:** 10.1038/s41598-022-14088-y

**Published:** 2022-06-21

**Authors:** Zhongjun Yang, Chuyan Dong, Xinyu Zhang, Guogang Wang

**Affiliations:** College of Information Engineering, Shenyang University of Chemical Technology, Shenyang, 110142 People’s Republic of China

**Keywords:** Electrical and electronic engineering, Mechanical engineering

## Abstract

We investigate the tracking control problem for a non-strict feedback nonlinear system with external disturbance and time-varying asymmetric full state constraints. Firstly, the unknown nonlinear term with external disturbance in the system are estimated by fuzzy logic system. The backstepping method is applied to the design of adaptive fuzzy controller. However, to prevent that the constraints are overstepped by introducing an improved log-type time-varying asymmetric barrier Lyapunov function (TABLF) in each step of backstepping design. Secondly, the dynamic surface control (DSC) is introduced in the designed algorithm to solve the computational explosion problem of controller caused by the derivative of control law. The proposed control scheme can speed up the tracking speed of the system. Compared with the previous work, it is verified that the combination of DSC and TABLF can obtain good performance within the constraint range, and can ensure fast and stable tracking convergence under external disturbance. Finally, two simulation examples verify the performance of the adaptive controller.

## Introduction

In the actual process of production, a majority of production equipment is a nonlinear system that affected by some uncertain factors such as parameter changes and external disturbances^1^. In recent years, researchers have proposed many effective ways to weaken these adverse impact. To be specific, the extensive application of both fuzzy logic systems (FLSs)^2^and neural networks (NNs)^3^ have been used to estimate unknown terms of the system by the use of adaptive backstepping technology^4^. Therefore, it is not difficult to know that the product of the combination of adaptive backstepping technology and FLS greatly solve the control problem of uncertain nonlinear system. At the same time, it can greatly improve system robustness.

From the other aspect of research, many concerns arise about constraint problems and a majority of actual systems operate under certain constraint conditions. For example, when the industrial manipulator is working, in order to make the manipulator move within the specified intervals, it is necessary to limit the rotation angle of the manipulator to avoid collision accidents. Therefore, it is important to deal with the constraint system. In recent years, the traditional Lyapunov functions do not have the ability to constrain the system state, therefore, by the positive impact of barrier Lyapunov function (BLF) on constraint properties of the viable, the state of system can be effectively kept in a specified scale and constraint control problem can thus be well solved. The control scheme based on BLF has been put forward continuously. The references^5–10^applies the BLF to realize the constraint control of the nonlinear systems. The reference^5^ combines BLF with preset performance control to control pure feedback nonlinear system, and constrains the state and tracks error of the system to a specified interval. The reference^6^ introduces BLF to the design process of the nonlinear system controller in the adaptive backstepping design method, which constrains the state of the system. The reference^7^ introduces a nonlinear state-dependent function constructed by coordinate transformation to eliminate constraints. In the practical application of restriction control, the references^8,9^ uses BLF to restrict the speed and current of the permanent magnet synchronous motor to ensure the safe operation of the motor. The reference^10^ applies BLF to restrict ship’s parameters such as ship’s lateral position, longitudinal position and heading. Compared with the traditional backstepping control method, the tracking errors converge on a small neighborhood of the origin and the full state constraints are not violated.

The above BLF-based constraint controls are time-invariant and symmetrical. However, in the actual industrial production system, there exists a situation that the constraint interval of the system state needs to be changed at any time during the different production progress, and the constraint interval is asymmetric. In this case, the time-varying asymmetric barrier Lyapunov function(TABLF) is required to constrain the system state with time-varying asymmetry. TABLF provides more flexibility in dealing with state constraints. Constraint control methods based on TABLF have received wide attention in recent years. The reference^11^ designs a robust adaptive controller for nonlinear systems with dynamic characteristics based on the TABLF, which limits the system output to the specified range. The reference^12^ applies TABLF to impose a time-varying asymmetric constraint on the full state of the input unmodeled dynamics system. The reference^13^ applies tan-type BLF working for both constrained and unconstrained scenarios to constrain all states of the nonlinear system with time-varying asymmetry. In addition to the common logarithmic BLF, there are integral BLF and tan-type BLF. Different BLF have their own characteristics and scope of application. Different types of BLF can be selected according to the control conditions. The TABLF has also made many achievements in practical application. The reference^14^ is combined with the finite-time stability theory, the log-type BLF is constructed to constrain state variables such as angular speed and stator current of permanent magnet synchronous motor in a predefined compact set. The reference^15^ uses TABLF to improve the control accuracy of aircraft. The reference^16^ uses asymmetric integral barrier Lyapunov functions are adopted to handle the fact that the operating regions of flight state variables are asymmetric in practice, while ensuring the validity of fuzzy-logic approximators. The reference^17^ applies log-type TABLF are utilized to confine flight states within some predefined compact sets all the time provided. System state constraint is a problem that must be carefully considered in the actual system. The constraint control for nonlinear systems is worth further studying.

Inspired by previous work, in comparison with the strict feedback systems and pure feedback systems, the non-strict feedback systems have more applicability in practical application. However, the traditional backstepping method can not be directly applied in the non-strict feedback systems. For this problem, the reference^18^ uses the method of variables separation to design the controller and provided a solution to the adaptive control problem of the non-strict feedback nonlinear systems. Compared with the variable separation method, the control method proposed in this paper removes the limitation of the unknown functions $\left| f_{i}(x)\right| \le {\Phi }(|x|)$ in references^18,19^, making the new method more widely applicable. However, the repeated differentiation in backstepping will result in the requirement of high-order differentiability and the complexity of controllers in the multiple-state high-order systems. This study introduces dynamic surface control (DSC) to deal with these problems. The controller constructed by backstepping DSC method is much simpler and has been well studied to solve the asymptotical tracking problem of non-strict feedback nonlinear systems. In recent years, many experts and scholars have applied the DSC method^14,20–24^ to solve the problem of computational complexity. The reference^14^ proposes an adaptive fuzzy finite-time DSC method for PMSM with full-state constraints. The reference^22^ introduces DSC to handle constraints for a class of nonlinear systems. The introduction of DSC technology further optimizes the design process of the adaptive backstepping control method, making it easier to design an adaptive controller for a nonlinear system.

Therefore, this paper presents a class of full state time-varying asymmetric constraints for non-strict feedback nonlinear system. It is different from strict feedback system and pure feedback system^25–28^. Firstly, an adaptive fuzzy controller for non-strict feedback systems is designed by using the adaptive backstepping method. TABLF is introduced in the design process to set the lower and upper bounds of the system state, thus, the full state time-varying asymmetric constraint of the system is realized. Secondly, by introducing DSC technology in the adaptive backstepping design process. The first-order filter is used to process the virtual control function, which solves the problem of repeated differential technology and reduces the computational complexity.

According to the above control methods, the main contributions and advantages of this paper are summarized as follows:

1. Different from the references^9,25–29^ that only focuses on the state constraints of strict feedback systems, this paper proposes a adaptive fuzzy control scheme considering full state constraints is investigated for non-strict feedback nonlinear systems and removes the limitation of the unknown functions $\left| f_{i}(x)\right| \le {\Phi }(|x|)$ in references^18,19^.
2. Compared with time-invariant symmetric constraint in references^30–32^, an improved TABLF method is used to solve time-varying asymmetric constraint control for non-strict feedback systems. And the DSC is introduced in the design process, which is used to reduce the order of TABLF, thus simplifying the design process of the controller.

## Problem formulation

### System description

Consider the following SISO non-strict feedback nonlinear system, an adaptive fuzzy controned to realize the full state time-varying asymmetric constraints of the system.

1 $$\left\{ \begin{array}{l} \dot{x}_{1} =f_{i}\left( \bar{x}_{n}\right) +x_{i+1}+\varepsilon _{i}\left( \bar{x}_{n}, t\right) \\ \dot{x}_{n} =f_{n}\left( \bar{x}_{n}\right) +u+\varepsilon _{n}\left( \bar{x}_{n}, t\right) \\ y =x_{1} \end{array}\right. $$

where $\bar{x}_{i}=\left[ x_{1}, x_{2}, \cdots , x_{i}\right] ^{T} \in R^{i}$ represents state vector, $f_{i}\left( \bar{x}_{n}\right) , i=1,2, \cdots , n$ denotes unknown smooth nonlinear function. $y\in R$ and $u\in R$ are the output and input of the system, respectively. $\varepsilon _{i}\left( \bar{x}_{n}, t\right)$ is the external disturbance, and $\varepsilon _{i}\left( \bar{x}_{n}, t\right)$ satisfies $\left| \bar{\varepsilon }_{i}\left( \bar{x}_{n}, t\right) \right| \le \bar{\varepsilon }_{i}$, $\varepsilon _{i}$ is a positive constant.

#### Assumption 1

Ref.^24^ It is assumed that the controlled system (1) is controllable and observable.

#### Remark 1

The system (1) is a class of non-strict feedback nonlinear systems with external disturbances. The non-strict feedback system in (1) is usually applied to the study of adaptive control, such as in references^33–35^. The one-link manipulator^36–39^ can be expressed in the form of the system.

The control objectives of this paper:

1. All signals in the closed-loop systems are bounded.
2. The system state does not violate the constraint conditions.
3. The tracking error of the system can remain within a prescribed constraint interval.

#### Assumption 2

For the lower and upper bounds $\bar{k}_{c i}(t)$ and $\underline{k}_{c i}(t)$ of the time-varying asymmetric constraint intervals, There exist the constants $\bar{K}_{c i}$ ,$\underline{K}_{c i}$, $\underline{D}_{c i j}$, $\bar{D}_{c i j}$, $i, j=1,2, \cdots n$ such that $\bar{k}_{c i}(t) \le \bar{K}_{c i}$, $\underline{k}_{c i}(t) \ge \underline{K}_{c i}$ and $\left| \bar{k}_{c i}^{j}(t)\right| \le \bar{D}_{c i j}$ and $\left| \underline{k}_{c i}^{j}(t)\right| \le \underline{D}_{c i j}$, where $\bar{k}_{c i}^{j}(t)$ and $\underline{k}_{c i}^{j}(t)$ denote $j-t h$ time derivative of $\bar{K}_{c i}$ and $\underline{K}_{c i}$.

#### Assumption 3

For reference signal $y_{r}(t)$ and its derivatives $y_{r}^{(k)}(t)$, there exist the functions $\bar{Y}_{0}(t): R_{+} \rightarrow R_{+}$, $\underline{Y}_{0}(t): R_{+} \rightarrow R_{+}$ satisfies $\overline{Y_{0}}(t)<\bar{k}_{c 1}(t)$, $\underline{Y_{0}}(t)<\underline{k}_{c 1}(t)$, and there also exist some positive parameters $Y_{1}, \cdots , Y_{n}$, such that $\underline{Y}_{0}(t) \le y_{r}(t) \le \bar{Y}_{0}(t)$, $\left| y_{r}^{(k)}(t)\right| \le Y_{k}$, $k=1,2, \cdots n$.

#### Remark 2

In order to meet the system control request, the above assumptions need to be made. Assumption 2 and 3 ensure that the lower and upper bounds of the constraint, the reference signal and its derivatives are all bounded, so that the functions involved in the derivation are bounded. The above assumptions are often used in the research of constrained control of nonlinear systems. For example, there are similar assumptions in reference^40^.

#### Assumption 4

The lumped uncertainties and external disturbance $f_{i}(\cdot )$ satisfy the linearly parameterizable condition: there exist uncertain vector $\theta ^{T}=\left[ \bar{y}_{1}, \bar{y}_{2}, \cdots , \bar{y}_{N}\right] =\left[ \theta _{1}, \theta _{2}, \cdots , \theta _{N}\right]$ and known smooth functions $\varphi (x)=\left[ \varphi _{1}(x), \varphi _{2}(x), \cdots , \varphi _{N}(x)\right] ^{T}$ such that $f_{i}\left( \bar{x}_{i}\right) =\theta ^{T} \varphi _{i}\left( \bar{x}_{i}\right)$.

#### Lemma 1

Ref.^41^ On account of the unknown function, we draw into the unknown function of FLS to approximate it. The form of function can be described as follows:

2 $$ \sup _{x \in \Omega } \mid f(x)-\theta ^{T}(\varphi (x) \mid \le \varepsilon $$

### The log-type TABLF construction

In the controller design process in this paper, all states of the nonlinear system are constrained to a specified interval by the BLF. The log-type TABLF construction can make the selection of the constraining interval of the system more flexible and can satisfy the constraining requirements of actual systems.

#### Definition 1

For the nonlinear system $\dot{x}=f(x)$, the smooth positive definite function *V*(*x*) is defined on the interval*U* containing the origin. Within interval *U*, *V*(*x*) has a first-order continuous partial derivative. If *X* approaches the boundary of interval *U*,$V(x) \rightarrow \infty$, $\forall t \in [0, \infty )$, $V(x) \le b$ and $b>0$ is constant when $x(0) \in U$. Then it is the BLF. The essence of the log-type TABLF is still BLF.

#### Lemma 2

Ref.^42^ For any positive constant $k_{b i}$, when ${e}_{i}$ satisfies $\left| e_{i}\right| <k_{b i}$, there are the following inequality:

3 $$ \log \frac{k_{b i}^{2}}{k_{b i}^{2}-e_{i}^{2}}<\frac{e_{i}^{2}}{k_{b i}^{2}-e_{i}^{2}} $$

#### Lemma 3

Ref.^43^ Considering the nonlinear system *f*(*x*), for smooth positive definite function *V*(*x*), if there exist scalars $\lambda >0$ and $\mu >0$, such that

4 $$ \dot{V}(x) \le -\lambda V+\mu $$

Then the solution of the nonlinear system is uniformly bounded.

#### Lemma 4

Ref.^44^ Let $k_{a}(t)$ and $k_{b}(t)$ be arbitrary functions, $Z=\left\{ e \in R:-k_{a}<e<k_{b}\right\} \subset R$ and $N=R^{l} \times Z \cup R^{n+1}$ are open sets. For the system (1), it is assumed that there are continuously differentiable positive definite functions $V: Z \rightarrow R^{+}$ and $U: R^{l} \rightarrow R^{+}$ such that

5 $$ \zeta _{1}(\Vert v\Vert ) \le W(v) \le \zeta _{2}(\Vert v\Vert ) $$

where $\zeta _{1}$ and $\zeta _{2}$ are $k_{\infty }$ type functions.

Let $V(\zeta )=V(\mathrm {e})+W(v)$, $e(0) \in Z$, if the following inequality is satisfied:

6 $$ \dot{V}=\frac{\partial v}{\partial \zeta } h \le -c V+\varepsilon $$

where $c>0$ and $\varepsilon <0$ are constants, then $e(t) \in Z, \forall t \in [0, \infty )$.

In order to impose time-varying asymmetric constraints on all states, the TABLF in references^44^ is introduced at each step of the controller design process

7 $$ \bar{V}_{i}=\frac{1-q\left( e_{i}\right) }{2 p} \log \left( \frac{k_{a i}^{2 p}(t)}{k_{a i}^{2 p}(t)-e_{i}^{2 p}(t)}\right) +\frac{q\left( e_{i}\right) }{2 p} \log \left( \frac{k_{a i}^{2 p}(t)}{k_{\infty }^{2 p}(t)-e_{i}^{2 p}(t)}\right) $$

where

$$ q(\cdot )=\left\{ \begin{array}{ll} 1, & \text{ if } >0 \\ 0, & \text{ if } <0 \end{array}\right. $$

It can be seen from (6) that the TABLF is a piecewise, continuous differentiable, positive definite function. The asymmetric BLF can design the lower and upper intervals of the constraint interval respectively. Compared with the symmetric BLF, it has more flexibility and wider application range, but the design process of the controller is also more difficult. Symmetric constant BLF can be regarded as a special case of (6), that is, the constraint interval is constant and symmetric up and down.

## Controller design

In order to design the controller, define the error variables as follows:

8 $$ \left\{ \begin{array}{l} e_{1}=x_{1}-y_{r} \\ e_{i}=x_{i}-\hat{\alpha }_{i-1} \\ e_{n}=x_{n}-\hat{\alpha }_{n-1} \end{array}\right. $$

The backstepping design process of the adaptive controller is as follows

Step 1: According to the system (1) and the defined error (8), we obtain

9 $$ \dot{e}_{1}=\dot{x}_{1}-\dot{y}_{r}=f_{1}\left( \bar{x}_{n}\right) +x_{2}+\varepsilon _{1}\left( \bar{x}_{n}, t\right) -\dot{y}_{r} $$

Then the introduction of first-order filter with a time constant $\tau _{1}$ has been used for virtual function.

10 $$ \tau _{1} \dot{\hat{\alpha }}_{1}+\hat{\alpha }_{1}=\alpha _{1}, \hat{\alpha }_{1}(0)=\alpha _{1}(0) $$

Thus, we could obtain the first-order filter error

11 $$ \chi _{1}=\hat{\alpha }_{1}-\alpha _{1} $$

Further we can get that

12 $$ \dot{\hat{\alpha }}_{1}=-\frac{\chi _{1}}{\tau _{1}} $$

According to (8), we can get

13 $$ x_{2}=e_{2}+\hat{\alpha }_{1} $$

Substituting (11) and (13) into (9), it can be written as

14 $$ \dot{e}_{1}=f_{1}\left( \bar{x}_{n}\right) +e_{2}+\chi _{1}+\alpha _{1}+\varepsilon _{1}(x, t)-\dot{y}_{r} $$

Then, we choose the TABLF candidate combined with quadratic Lyapunov function as

15 $$ V_{1}=\frac{1-q\left( e_{1}\right) }{2 p} \log \left( \frac{k_{a 1}^{2 p}(t)}{k_{a 1}^{2 p}(t)-e_{1}^{2 p}(t)}\right) +\frac{q\left( e_{1}\right) }{2 p} \log \left( \frac{k_{b 1}^{2 p}(t)}{k_{b 1}^{2 p}(t)-z_{1}^{2 p}(t)}\right) +\frac{\tilde{\theta }_{1}^{2}}{2 \zeta _{1}}+\frac{\chi _{1}^{2}}{2} $$

where

$$\begin{aligned} q\left( e_{1}\right) =\left\{ \begin{array}{ll} 1, & e_{1}>0 \\ 0, & e_{1}<0 \end{array}\right. \end{aligned}$$

where $\zeta_{1}$ is a positive design parameter, $\theta _{1}$ denotes the estimation of $\theta_{1}^{*}$ , $\tilde{\theta }_{1}=\theta _{1}^{*}-\theta _{1}$ stands for the estimation error.

The time-varying constraints $k_{a 1}(t)$ and $k_{b 1}(t)$ on output tracking error $e_{1}$ in (15) corresponding to output constraints $\underline{k}_{c 1}(t)$, $\bar{k}_{c 1}$ are given by

16 $$ k_{a 1}(t)=y_{r}(t)-\underline{k}_{c 1}(t),k_{b 1}(t)=\bar{k}_{c 1}(t)-y_{r}(t) $$

By Assumptions 2 and 3, there exist positive constants $\underline{K}_{a 1}(t)$, $\bar{K}_{a 1}$, $\underline{K}_{b 1}(t)$, $\bar{K}_{b 1}$ such that $\underline{K}_{a 1} \le k_{a 1}(t) \le \bar{K}_{a 1}$, $\underline{K}_{a 1} \le k_{b 1}(t) \le \bar{K}_{b 1}$, $\forall \ge 0$.

The derivative of $V_{1}$ is given by

17 $$\begin{aligned}  \dot{V}_{1}=\,&e_{1} K_{e 1}\left[ f_{1}\left( \bar{x}_{n}\right) +e_{2}+\chi _{1}+\alpha _{1}+\varepsilon _{1}\left( \bar{x}_{n}, t\right) -\dot{y}_{r}\right. \\&\left. +\left( 1-q\left( e_{1}\right) \right) \frac{\dot{k}_{a 1}(t)}{k_{a 1}(t)} e_{1}+\left( q\left( e_{1}\right) \right) \frac{\dot{k}_{b 1}(t)}{k_{b 1}(t)} e_{1}\right] \\&-\frac{\tilde{\theta }_{1} \dot{\theta }_{1}}{\zeta _{1}}+\chi _{1}\left( -\chi _{1} / \tau _{1}-\dot{\alpha }_{1}\right) \end{aligned}$$

where

$$ K_{e 1}=\frac{1-q\left( e_{1}\right) }{k_{a 1}^{2}(t)-e_{1}^{2}}+\frac{q\left( e_{1}\right) }{k_{b 1}^{2}(t)-e_{1}^{2}} $$

According to Lemma 1, we can have

18 $$ f_{1}\left( \bar{x}_{n}\right) =\Theta _{1}^{T} \varphi _{1}\left( \bar{x}_{n}\right) +\lambda _{1}\left( \bar{x}_{n}\right) ,\lambda _{1}\left( \bar{x}_{n}\right) \le \bar{\lambda }_{1} $$

where $$\forall \overline{\lambda _{1}}>0$$.

Since $0<\varphi _{i} \varphi _{i}^{T}<1$, $i=1,2, \cdots , n$ the following inequalities can be obtained

19 $$\begin{aligned} e_{1} K_{e 1} \Theta _{1}^{T} \varphi _{1}\left( \bar{x}_{n}\right)&\le \frac{e_{1}^{2} K_{e 1}^{2}\left[ \Theta _{1}^{T} \varphi _{1}\left( \bar{x}_{n}\right) \right] ^{2}}{2 \omega _{1}^{2}}+\frac{\omega _{1}^{2}}{2}\nonumber \\&\le \frac{e_{1}^{2} K_{e 1}^{2} \Theta _{1}^{T} \Theta _{1} \varphi _{1}^{T}\left( \bar{x}_{n}\right) \varphi _{1}\left( \bar{x}_{n}\right) }{2 \omega _{1}^{2}}+\frac{\omega _{1}^{2}}{2}\nonumber \\&\le \frac{{\kappa _{1}} e_{1}^{2} K_{e 1}^{2} \theta _{1}^{*} \psi _{1}^{T}\left( \bar{x}_{n}\right) \varphi _{1}\left( \bar{x}_{n}\right) }{2 \omega _{1}^{2}}+\frac{\omega _{1}^{2}}{2}\nonumber \\&\le \frac{{\kappa _{1}} e_{1}^{2} K_{e 1}^{2} \theta _{1}^{*} \varphi _{1}^{T}\left( \bar{x}_{n}\right) \varphi _{1}\left( \bar{x}_{n}\right) }{2 \omega _{1}^{2} \varphi _{1}^{T}\left( x_{1}\right) \varphi _{1}\left( x_{1}\right) }+\frac{\omega _{1}^{2}}{2}\nonumber \\&\le \frac{ {\kappa _{1}} e_{1}^{2} K_{e 1}^{2} \theta _{1}^{*}}{2 \omega _{1}^{2} \varphi _{1}^{T}\left( x_{1}\right) \varphi _{1}\left( x_{1}\right) }+\frac{\omega _{1}^{2}}{2} \end{aligned}$$

20 $$ e_{1} K_{e 1} \lambda _{1}(x) \le \frac{{\kappa _{1}} e_{1}^{2} K_{e 1}^{2}}{2 n_{1}^{2}}+\frac{n_{1}^{2} \bar{\lambda }_{1}^{2}}{2 {\kappa _{1}}}$$

21 $$ e_{1} K_{e 1} \varepsilon _{1}(x, t) \le \frac{e_{1}^{2} K_{e 1}^{2}}{2}+\frac{\bar{\varepsilon }_{1}^{2}}{2} $$

22 $$ e_{1} \chi _{1} \le e_{1}^{2}+\frac{\chi _{1}^{2}}{4} $$

where $\theta _{1}^{*}=\frac{\left\| \Theta _{1}\right\| ^{2}}{\kappa _{1}}$, $\omega _{1}$, ${\kappa _{1}}$ and $\eta _{1}$ are positive design parameters.

By substituting (18)-(22) into (17), the following inequality can be obtained:

23 $$  \begin{aligned}    & \dot{V}_{1}  \le   e_{1} K_{{e1}} \left[ {\frac{{\kappa _{1} e_{1} K_{{e1}} \theta _{1}^{*} }}{{2\omega _{1}^{2} \varphi _{1}^{T} \left( {x_{1} } \right)\varphi _{1} \left( {x_{1} } \right)}} + } \right.\frac{{\kappa _{1} e_{1} K_{{e1}} }}{{2n_{1}^{2} }} + \frac{{e_{1} K_{{e1}} }}{2} - \dot{y}_{r}  + e_{2}  \\     &\quad  + \alpha _{1} \left. { + \left( {1 - q\left( {e_{1} } \right)} \right)\frac{{\dot{k}_{{a1}} (t)}}{{k_{{a1}} (t)}}e_{1}  + \left( {q\left( {e_{1} } \right)} \right)\frac{{\dot{k}_{{b1}} (t)}}{{k_{{b1}} (t)}}e_{1} } \right] + \frac{{\omega _{1}^{2} }}{2} + \frac{{\eta _{1}^{2} \bar{\lambda }_{1}^{2} }}{{2\kappa _{1} }} \\     & \quad  + \frac{{\bar{\varepsilon }_{1}^{2} }}{2} - \frac{{\tilde{\theta }_{1} \dot{\theta }_{1} }}{{\zeta _{1} }} + \chi _{1} \left( { - \frac{{\chi _{1} }}{{\tau _{1} }} - \dot{\alpha }_{1} } \right) + \frac{{\chi _{1}^{2} K_{{e1}} }}{4} + e_{1}^{2} K_{{e1}}  \\  \end{aligned}  $$

Select the virtual control function $\alpha _{1}$ and adaptive law $\dot{\theta }_{1}$ as

24 $$ \alpha _{1}=-\left( \sigma _{1}+\nu _{1}(t)\right) e_{1}-\frac{\kappa _{1} e_{1} K_{e 1} \theta _{1}}{2 \omega _{1}^{2} \varphi _{1}^{T}\left( x_{1}\right) \varphi _{1}\left( x_{1}\right) }-\frac{\kappa _{1} e_{1} K_{e 1}}{2 \eta _{1}^{2}}-\frac{e_{1} K_{e 1}}{2}+\dot{y}_{r} $$

25 $$ \dot{\theta }_{1}=\frac{\zeta _{1} k_{1} e_{1}^{2} K_{e 1}^{2}}{2 \omega _{1}^{2} \varphi _{1}^{T}\left( x_{1}\right) \varphi _{1}\left( x_{1}\right) }-\gamma _{1} \theta _{1} $$

where $\sigma _{1}>0$ and $\gamma _{1}>0$ are design parameters, and the time-varying gain is given $v_{1}(t)$by

26 $$ v_{1}(t)=\sqrt{\left( 1-q\left( e_{1}\right) \right) \left( \frac{{k}_{a 1}}{k_{a 1}}\right) ^{2}+q\left( e_{1}\right) \left( \frac{\dot{k}_{b 1}}{k_{b 1}}\right) ^{2}+\zeta } $$

Under the Assumptions 2 and 3, we concluded that $x_{1}$, $y_{r}$, $\dot{y}_{r}$, $k_{a 1}$, $\dot{k}_{a l}$, $k_{b 1}$, $\dot{k}_{b 1}$ are continuous and bounded with a maximum absolute value $A_{1}$. According to Young’s inequality, we have:

27 $$ \left| \chi _{1} \dot{\alpha }_{1}\right| \le \frac{\chi _{1}^{2} A_{1}^{2}}{2 \iota _{1}^{2}}+\frac{\iota _{1}^{2}}{2} $$

According to (24), (25) and (27), (23) can be written as

28 $$ \begin{aligned} \dot{V}_{1} \le&-\left( \sigma _{1}-1\right) e_{1}^{2} K_{e 1}+e_{1} e_{2} K_{e 1}+\frac{\omega _{1}^{2}}{2}+\frac{\eta _{1}^{2} \bar{\lambda }_{1}^{2}}{2 {\kappa _{1}}} \\&+\frac{\overline{\varepsilon _{1}}^{2}}{2}+\frac{\gamma _{1} \theta _{1} \tilde{\theta }_{1}}{\zeta _{1}}-\chi _{1}^{2}\left[ \frac{1}{\tau _{1}}-\frac{K_{e 1}}{4}-\frac{A_{1}^{2}}{2 t_{1}^{2}}\right] +\frac{{\iota _{1}}^{2}}{2} \end{aligned}$$

where

$$ \frac{\gamma _{1} \tilde{\theta }_{1} \theta _{1}}{\zeta _{1}}=\frac{\gamma _{1} \tilde{\theta }_{1}\left( \theta _{1}^{*}-\tilde{\theta }_{1}\right) }{\zeta _{1}} \le \frac{\gamma _{1} \theta _{1}^{* 2}}{2 \zeta _{1}}-\frac{\gamma _{1} \tilde{\theta }_{1}^{2}}{2 \zeta _{1}} $$

then (28) can be further expressed as

29 $$ \begin{aligned} \dot{V}_{1} \le&-\left( \sigma _{1}-1\right) e_{1}^{2} K_{e 1}+e_{1}e_{2} K_{e 1}-\frac{\gamma _{1} \tilde{\theta }_{1}^{2}}{2 \zeta _{1}}\\&-\chi _{1}^{2}\left[ \frac{1}{\tau _{1}}-\frac{K_{e 1}}{4}-\frac{A_{1}^{2}}{2 \iota ^{2}}\right] +\frac{\bar{\varepsilon }_{1}^{2}}{2}+\frac{\omega _{1}^{2}}{2}\\&+\frac{{\varepsilon _{1}}^{2} \bar{\lambda }_{1}^{2}}{2 \Sigma _{1}}+\frac{\gamma _{1} {\varepsilon }_{1}^{* 2}}{2 \zeta _{1}}+\frac{{\iota _{1}}^{2}}{2} \end{aligned}$$

Therefore, the selection range of constant gain and $\sigma _{1}$ time constant $\tau _{1}$ should be limited to $\frac{1}{\tau _{1}} \ge \frac{K_{e 1}}{4}+\frac{A_{1}^{2}}{2 \tau _{1}^{2}}$ and in order to guarantee the closed-loop stability.

Step i $(i=2,3, \cdots , n-1)$: According to the system (1) and the defined error (8), we obtain

30 $$ \dot{e}_{i}=\dot{x}_{i}-\dot{\hat{\alpha }}_{i-1}=f_{i}\left( \bar{x}_{n}\right) +x_{i+1}+\varepsilon _{i}\left( \bar{x}_{n}, t\right) -\dot{\hat{\alpha }}_{i-1} $$

Then the introduction of first-order filter with a time constant has $\tau _{i}$ been used for virtual function $\alpha _{i}$.

31 $$ \tau _{i} \hat{\alpha }_{i}+\hat{\alpha }_{i}=\alpha _{i},\hat{\alpha }_{i}(0)=\alpha _{i}(0) $$

Thus, we could obtain the first-order filter error

32 $$ \chi _{i}=\hat{\alpha }_{i}-\alpha _{i} $$

We can further obtain that

33 $$ \dot{\hat{\alpha }}_{i}=\frac{-\chi _{i}}{\tau _{i}} $$

According to (8), we can get that

34 $$ \tilde{x}_{i+1}=\hat{e}_{i+1}+\hat{\alpha }_{i} $$

According to (36) and (38), (34) can be written as

35 $$ \dot{e}_{i}=f_{i}\left( \bar{x}_{n}\right) +e_{i+1}+\chi _{i}+\alpha _{i}+\varepsilon _{i}(x, t)-\dot{\hat{\alpha }}_{i-1} $$

Then, we choose the TABLF candidate combined with quadratic Lyapunov Function as

36 $$ \begin{aligned} V_{i}=&V_{i-1}+\frac{1-q\left( e_{i}\right) }{2} \log \left( \frac{k_{a i}^{2}(t)}{k_{a i}^{2}(t)-e_{i}^{2}(t)}\right) \\&+\frac{q\left( e_{i}\right) }{2} \log \left( \frac{k_{b i}^{2}(t)}{k_{b i}^{2}(t)-z_{i}^{2}(t)}\right) +\frac{\tilde{\theta }_{i}^{2}}{2 \zeta _{i}}+\frac{\chi _{i}^{2}}{2} \end{aligned}$$

where

$$ q\left( e_{i}\right) =\left\{ \begin{array}{ll} 1, & e_{i}>0 \\ 0, & e_{i}<0 \end{array}\right. $$

where $\zeta _{i}$ is a positive design parameter, $\theta _{i}$ denotes the estimation of $\theta _{i}^{*}$ , $\tilde{\theta }_{i}=\theta _{i}^{*}-\theta _{i}$ stands for the estimation error.

The time-varying constraints $k_{a i}(t)$ and $k_{b i}(t)$ on output tracking error $e_{i}$ in (15) corresponding to output constraints $\underline{k}_{c i}(t)$, $\bar{k}_{c i}$ are given by

37 $$ k_{a i}(t)=y_{r}(t)-\underline{k}_{c i}(t),k_{b i}(t)=\bar{k}_{c i}(t)-\alpha _{i-1}(t) $$

By Assumptions 2 and 3, there exist positive constants $\underline{k}_{a i}(t)$, $\bar{k}_{a i}$, $\underline{k}_{b i}(t)$, $\bar{k}_{b i}$ such that $\underline{K}_{a i} \le k_{a i}(t) \le \bar{K}_{a i}$, $\underline{K}_{a i} \le k_{b i}(t) \le \bar{K}_{b i}$, $\forall \ge 0$.

The derivative of ${V}_{i}$ , we can obtain that

38 $$ \begin{aligned} \dot{V}_{i}&=\dot{V}_{i-1}+e_{i} K_{e i}\left[ f_{i}\left( \bar{x}_{n}\right) +e_{i+1}+\chi _{i}+\alpha _{i}+\varepsilon _{i}\left( \bar{x}_{n}, t\right) -\dot{\hat{\alpha }}_{i-1}\right. \\&\left.\quad +\left( 1-q\left( e_{i}\right) \right) \frac{\dot{k}_{a i}(t)}{k_{a i}(t)} e_{i}+\left( q\left( e_{i}\right) \right) \frac{\dot{k}_{b i}(t)}{k_{b i}(t)} e_{i}\right] -\frac{\tilde{\theta }_{i} \dot{\theta }_{i}}{\zeta _{i}}+\chi _{i}\left( -\frac{\chi _{i}}{\tau _{i}}-\dot{\alpha }_{i}\right) \end{aligned} $$

where

$$ K_{e i}=\frac{1-q\left( e_{i}\right) }{k_{a^{2}}^{2}(t)-e_{i}^{2}}+\frac{q\left( e_{i}\right) }{k_{b i}^{2}(t)-e_{i}^{2}} $$

According to Lemma 1, we can have:

39 $$ f_{i}\left( \bar{x}_{n}\right) =\Theta _{i}^{T} \varphi _{i}\left( \bar{x}_{n}\right) +\lambda _{i}\left( \bar{x}_{n}\right) ,\lambda _{i}\left( \bar{x}_{n}\right) \le \bar{\lambda }_{i} $$

where $\lambda _{i}\left( \bar{x}_{n}\right) \le \bar{\lambda }_{i}$ and $\bar{\lambda }_{i}>0$ are constants.

By applying Young’s inequality, the following inequality can be obtained

40 $$\begin{aligned} e_{i} K_{e i} \Theta _{i}^{T} \varphi _{i}\left( \bar{x}_{n}\right)&\le \frac{e_{i}^{2} K_{e i}^{2}\left[ \Theta _{i}^{T} \varphi _{i}\left( \bar{x}_{n}\right) \right] ^{2}}{2 \omega _{i}^{2}}+\frac{\omega _{i}^{2}}{2} \nonumber \\&\le \frac{\kappa _{i} e_{i}^{2} K_{e i}^{2} \theta _{i}^{*} \varphi _{i}^{T}\left( \bar{x}_{n}\right) \varphi _{i}\left( \bar{x}_{n}\right) }{2 \omega _{2}^{2}}+\frac{\omega _{i}^{2}}{2} \nonumber \\&\le \frac{\kappa _{i} e_{i}^{2} K_{e i}^{2} \theta _{i}^{*} \varphi _{i}^{T}\left( \bar{x}_{n}\right) \varphi _{i}\left( \bar{x}_{n}\right) }{2 \omega _{i}^{2} \varphi _{i}^{T}\left( \bar{x}_{i}\right) \varphi _{i}\left( \bar{x}_{i}\right) }+\frac{\omega _{i}^{2}}{2} \nonumber \\&\le \frac{\kappa {K}_{i} e_{i}^{2} K_{e i}^{2} \theta _{i}^{*}}{2 \omega _{i}^{2} \varphi _{i}^{T}\left( \bar{x}_{i}\right) \varphi _{i}\left( \bar{x}_{i}\right) }+\frac{\omega _{i}^{2}}{2} \end{aligned}$$

41 $$ e_{i} K_{e i} \lambda _{i}(x) \le \frac{{K}_{i} e_{i}^{2} K_{e i}^{2}}{2 n_{i}^{2}}+\frac{\eta _{i}^{2} \bar{\lambda }_{i}^{2}}{2 k_{i}} $$

42 $$ e_{i} K_{e i} \varepsilon _{i}(x, t) \le \frac{e_{i}^{2} K_{e i}^{2}}{2}+\frac{\bar{\varepsilon }_{i}^{2}}{2} $$

43 $$ e_{i} \chi _{i} \le e_{i}^{2}+\frac{\chi _{i}^{2}}{4} $$

where $\theta _{i}^{*}=\frac{\left\| \Theta _{1}\right\| ^{2}}{k_{i}}$, $\omega _{1}$, ${k}_{i}$ and $\eta _{i}$ are positive design parameters.

According to the derivation process in the previous step, we can get that

44 $$\begin{aligned}  \dot{V}_{i-1} \le&-\sum _{k=1}^{i-1}\left( \sigma _{k}-1\right) e_{k}^{2} K_{e k}+e_{i-1} e_{i} K_{e i-1}-\sum _{k=1}^{i-1} \frac{\gamma _{k} \tilde{\theta }_{k}^{2}}{2 \zeta _{\mathrm {k}}} \\&-\sum _{k=1}^{i-1} \chi _{k}^{2}\left[ \frac{1}{\tau _{k}}-\frac{K_{e k}}{4}-\frac{A_{k}^{2}}{2 \iota _{k}^{2}}\right] +\sum _{k=1}^{i-1} \xi _{k} . \end{aligned} $$

Based on (39)–(44), (38) can be expressed as

45 $$ \begin{aligned} \dot{V}_{i} &\le-\sum _{k=1}^{i-1}\left( \sigma _{k}-1\right) e_{k}^{2} K_{e k}+e_{i-1} e_{i} K_{e i-1}-\sum _{k=1}^{i-1} \frac{\gamma _{k} \tilde{\theta }_{k}^{2}}{2 \zeta _{{\text {k}}}}-\sum_{k=1}^{i-1} \chi _{k}^{2}\left[ \frac{1}{\tau _{k}}-\frac{K_{e k}}{4}-\frac{A_{k}^{2}}{2 t_{k}^{2}}\right] \\ &\quad+\sum _{k=1}^{i-1} \xi _{k}+e_{i} K_{e i}\left[ \frac{{}_{i}/ e_{i} K_{e i} \theta _{i}^{*}}{2 \omega _{i}^{2} \varphi _{i}^{T}\left( \bar{x}_{i}\right) \varphi _{i}\left( \bar{x}_{i}\right) }+\frac{{}_{i}/ e_{i} K_{e i}}{2 \eta _{i}^{2}}+\frac{e_{i} K_{e i}}{2}+\frac{\chi _{i-1}}{\tau _{i-1}}+e_{i+1}+\alpha _{i}\right. \\ &\quad\left. +\left( 1-q\left( e_{i}\right) \right) \frac{\dot{k}_{a i}(t)}{k_{a i}(t)} e_{i}+\left( q\left( e_{i}\right) \right) \frac{\dot{k}_{b i}(t)}{k_{b i}(t)} e_{i}\right] +\frac{\omega _{i}^{2}}{2}+\frac{\eta _{i}^{2} \bar{\lambda }_{i}^{2}}{2 \kappa _{i}}+\frac{{\bar{\varepsilon }}_{i}^{2}}{2}-\frac{\tilde{\theta }_{i} \dot{\theta }_{i}}{\zeta _{i}}\\ &\quad+\chi _{i}\left( -\frac{\chi _{i}}{\tau_{i}}-\dot{\alpha }_{i}\right) +\frac{\chi_{i}^{2} K_{e i}}{4}+e_{i}^{2} K_{e i} \end{aligned} $$

Select the virtual control function $\alpha_{i}$ and adaptive law $\dot{\theta}_{i}$ as

46 $$ \alpha_{i}=-\left( \sigma _{i}+v_{i}(t)\right) e_{i}-\frac{K_{i} e_{i} K_{e i} \theta _{i}}{2 \omega _{i}^{2} \varphi _{i}^{T}\left( \bar{x}_{i}\right) \varphi _{i}\left( \bar{x}_{i}\right) }-\frac{\kappa _{i} e_{i} K_{e i}}{2 \eta _{i}^{2}}-\frac{e_{i} K_{e i}}{2}-\frac{\chi _{i-1}}{\tau _{i-1}}-\frac{K_{e i-1}}{K_{e i}} e_{{\text{i}}-1} $$

47 $$ \dot{\theta }_{i}=\frac{\zeta _{i} \kappa _{i} e_{i}^{2} K_{e i}^{2}}{2 \omega _{i}^{2} \varphi _{i}^{T}\left( \bar{x}_{i}\right) \varphi _{i}\left( \bar{x}_{i}\right) }-\gamma _{i} \theta _{i} $$

where $\sigma _{i}>0$ and $\gamma _{i}>0$ are design parameters, and the time-varying gain is given $v_{i}(t)$by

48 $$ v_{i}(t)=\sqrt{\left( 1 - q\left( e_{i}\right) \right) \left( \frac{\dot{k}_{a i}}{k_{a i}}\right) ^{2}+q\left( e_{i}\right) \left( \frac{\dot{k}_{b i}}{k_{b i}}\right) ^{2}+\zeta } $$

Using the analysis method in step 1, we can see that both $\dot{\alpha }_{i}$ and $\alpha _{i}$ are bounded, and there is a positive parameter $A_i$ that satisfies.

49 $$ \left| \chi _{i} \dot{\alpha }_{i}\right| \le \frac{\chi _{i}^{2} A_{i}^{2}}{2 \iota _{i}^{2}}+\frac{\iota _{i}^{2}}{2} $$

Substitute (46), (47) and (49) into (45) to obtain

50 $$ \begin{aligned} \dot{V}_{i} \le&-\sum _{k=1}^{i}\left( \sigma _{k}-1\right) e_{k}^{2} K_{e k}+e_{i} e_{i+1} K_{e i}-\sum _{k=1}^{i-1} \frac{\gamma _{k} \tilde{\theta }_{k}^{2}}{2 \zeta _{k}}-\sum _{k=1}^{i} \chi _{k}^{2}\left[ \frac{1}{\tau _{k}}-\frac{K_{e k}}{4}-\frac{A_{k}^{2}}{2 \iota _{k}^{2}}\right] \\&+\sum _{k=1}^{i-1} \xi _{k}+\frac{\omega _{i}^{2}}{2}+\frac{\eta _{i}^{2} \bar{\lambda }_{i}^{2}}{2 \kappa _{i}}+\frac{\bar{\varepsilon }_{i}^{2}}{2}+\frac{Y_{i} \theta _{i} \tilde{\theta }_{i}}{\zeta _{i}}+\frac{\iota _{i}^{2}}{2} \end{aligned}$$

where

$$ \frac{\gamma _{i} \tilde{\theta }_{i} \theta _{i}}{\zeta _{i}}=\frac{\gamma _{i} \tilde{\theta }_{i}\left( \theta _{i}^{*}-\tilde{\theta }_{i}\right) }{\zeta _{i}} \le \frac{\gamma _{i} \theta _{i}^{* 2}}{2 \zeta _{i}}-\frac{\gamma _{i} \tilde{\theta }_{i}^{2}}{2 \zeta _{i}} $$

Thus, (50) can be obtained

51 $$ \begin{aligned} \dot{V}_{i} \le&-\sum _{k=1}^{i}\left( \sigma _{k}-1\right) e_{k}^{2}{K}_{e k}{e}_{i} e_{i+1}{K}_{e i}-\sum _{k=1}^{i} \frac{\gamma _{k} \tilde{\theta }_{k}^{2}}{2 \xi _{k}} \\&-\sum _{k=1}^{i} {\chi }_{k}^{2}\left[ \frac{1}{{\tau }_{k}}-\frac{{K}_{e k}}{4}-\frac{{A}_{k}^{2}}{2 {\iota }_{k}^{2}}\right] +\sum _{k=1}^{i} {\xi }_{k}, \end{aligned} $$

where $$\xi _{i}=\frac{\overline{\varepsilon _{i}}^{2}}{2}+\frac{\omega _{i}^{2}}{2}+\frac{\eta _{i}^{2} \bar{\lambda }_{i}^{2}}{2 \kappa _{i}}+\frac{\gamma _{i} \theta _{i}^{* 2}}{2 \zeta _{i}}+\frac{\iota _{i}^{2}}{2}.$$

Therefore, the selection range of constant gain $\sigma _{i}$ and time constant $\tau _{i}$ should be limited to $\sigma _{1}>1$ and $\frac{1}{\tau _{i}} \ge \frac{K_{e i}}{4}+\frac{A_{i}^{2}}{2 \iota _{i}^{2}}$ in order to guarantee the closed-loop stability.

Step n: According to the system (1) and the defined error (8), we obtain the derivative of ${e}_{n}$

52 $$ \dot{e}_{n}=\dot{x}_{n}-\dot{\hat{\alpha }}_{n-1}=f_{n}\left( \bar{x}_{n}\right) +u+\varepsilon _{n}\left( \bar{x}_{n}, t\right) -\dot{\hat{\alpha }}_{n-1} $$

Then, we choose the TABLF candidate combined with quadratic Lyapunov Function as

53 $$ \begin{aligned} V_{n}=&V_{n-1}+\frac{1-q\left( e_{n}\right) }{2} \log \left( \frac{k_{a n}^{2}(t)}{k_{a n}^{2}(t)-e_{n}^{2}(t)}\right) \\&+\frac{q\left( e_{n}\right) }{2} \log \left( \frac{k_{b n}^{2}(t)}{k_{b n}^{2}(t)-z_{n}^{2}(t)}\right) +\frac{\tilde{\theta }_{n}^{2}}{2 \zeta _{n}} \end{aligned} $$

where

$$ q\left( e_{n}\right) =\left\{ \begin{array}{ll} 1, & e_{n}>0 \\ 0, & e_{n}<0 \end{array}\right. $$

where $\zeta _{n}$ is a positive design parameter, $\theta _{n}$ denotes the estimation of ${\theta _{n}^{*}}$, $\tilde{\theta }_{n}={\theta _{i}^{*}}-\theta _{n}$ stands for the estimation error.

The time-varying constraints $k_{a n}(t)$ and $k_{b i}(t)$ on output tracking error $e_{n}$ in (15) corresponding to output constraints $\underline{k}_{c n}(t)$, $\bar{k}_{c i}$ are given by

54 $$ k_{a i}(t)=y_{r}(t)-\underline{k}_{c i}(t),k_{b i}(t)=\bar{k}_{c i}(t)-\alpha _{n-1}(t) $$

By Assumptions 2 and 3, there exist positive constants $\underline{K}_{a n}(t)$, $\bar{K}_{a i}$, $\underline{K}_{b n}(t)$,$\bar{K}_{b i}$ such that $\underline{K}_{a i} \le k_{a i}(t) \le \bar{K}_{a i}$, $\underline{K}_{a n} \le k_{b i}(t) \le \bar{K}_{b i}$, $\forall \ge 0$.

According to (52) and (53), we can get that

55 $$ \begin{aligned} \dot{V}_{n}=&\dot{V}_{n-1}+e_{n} K_{e i}\left[ f_{n}\left( \bar{x}_{n}\right) +e_{n+1}+\chi _{n}+\alpha _{n}+\varepsilon _{n}\left( \bar{x}_{n}, t\right) -\dot{\hat{\alpha }}_{i-1}\right. \\&\left. +\left( 1-q\left( e_{n}\right) \right) \frac{\dot{k}_{a n}(t)}{k_{a n}(t)} e_{n}+\left( q\left( e_{n}\right) \right) \frac{\dot{k}_{b n}(t)}{k_{b n}(t)} e_{n}\right] -\frac{\tilde{\theta }_{n} \dot{\theta }_{i}}{\zeta _{n}} \end{aligned} $$

where

$$ K_{e n}=\frac{1-q\left( e_{n}\right) }{k_{a n}^{2}(t)-e_{n}^{2}}+\frac{q\left( e_{n}\right) }{k_{b n}^{2}(t)-e_{n}^{2}} $$

From step n-1 of the derivation process, we can get that

56 $$ \begin{aligned} \dot{V}_{n-1} \le&-\sum _{k=1}^{n-1}\left( \sigma _{k}-1\right) e_{k}^{2} {K}_{e k}+{e}_{n-1} e_{n} {K}_{e n-1}-\sum _{k=1}^{n-1} \frac{\gamma _{k} \tilde{\theta }_{k}^{2}}{2 \xi _{k}} \\&-\sum _{k=1}^{n-1} {\chi }_{k}^{2}\left[ \frac{1}{{\tau }_{k}}-\frac{{K}_{e k}}{4}-\frac{{A}_{k}^{2}}{2 {t}_{k}^{2}}\right] +\sum _{k=1}^{n-1} {\xi }_{k}, \end{aligned} $$

According to Lemma 1, we can have :

57 $$ f_{n}\left( \bar{x}_{n}\right) =\Theta _{n}^{T} \varphi _{n}\left( \bar{x}_{n}\right) +\lambda _{n}\left( \bar{x}_{n}\right) ,\lambda _{n}\left( \bar{x}_{n}\right) \le \bar{\lambda }_{n} $$

By applying Young’s inequality, the following inequality can be obtained

58 $$\begin{aligned} e_{n} K_{e n} \Theta _{n}^{T} \varphi _{n}\left( \bar{x}_{n}\right)&\le \frac{e_{n}^{2} K_{e n}^{2}\left[ \Theta _{n}^{T} \varphi _{n}\left( \bar{x}_{n}\right) \right] ^{2}}{2 \omega _{n}^{2}}+\frac{\omega _{n}^{2}}{2} \nonumber \\&\le \frac{\kappa _{n} e_{n}^{2} K_{e n}^{2} \theta _{n}^{*} \varphi _{n}^{T}\left( \bar{x}_{n}\right) \varphi _{n}\left( \bar{x}_{n}\right) }{2 \omega _{2}^{2}}+\frac{\omega _{n}^{2}}{2} \nonumber \\&\le \frac{\kappa _{n} e_{n}^{2} K_{e n}^{2} \theta _{n}^{*} \varphi _{n}^{T}\left( \bar{x}_{n}\right) \varphi _{n}\left( \bar{x}_{n}\right) }{2 \omega _{i}^{2} \varphi _{n}^{T}\left( \bar{x}_{n}\right) \varphi _{i}\left( \bar{x}_{n}\right) }+\frac{\omega _{n}^{2}}{2} \nonumber \\&\le \frac{\kappa _{n} e_{n}^{2} K_{e n}^{2} \theta _{n}^{*}}{2 \omega _{n}^{2} \varphi _{n}^{T}\left( \bar{x}_{n}\right) \varphi _{n}\left( \bar{x}_{n}\right) }+\frac{\omega _{n}^{2}}{2}\end{aligned}$$

59 $$ e_{n} K_{e n} \lambda _{n}(x) \le \frac{\kappa _{n} e_{n}^{2} K_{e n}^{2}}{2 n_{n}^{2}}+\frac{\eta _{n}^{2} \bar{\lambda }_{n}^{2}}{2 k_{n}} $$

60 $$ e_{n} K_{e n} \varepsilon _{i}(x, t) \le \frac{e_{n}^{2} K_{e n}^{2}}{2}+\frac{\bar{\varepsilon }_{n}^{2}}{2} $$

where $\theta _{n}^{*}=\frac{\left\| \Theta _{n}\right\| ^{2}}{k_{n}}$, $\omega _{1}$, ${\kappa }_{n}$ and $\eta _{n}$ are positive design parameters.

Substituting the (57)-(60) into (55), so that

61 $$ \begin{aligned} \dot{V}_{n} \le&-\sum _{k=1}^{i}\left( \sigma _{k}-1\right) e_{k}^{2} K_{e k}+e_{n-1} e_{n} K_{en-1}-\sum _{k=1}^{n-1} \frac{\gamma _{k} \tilde{\theta }_{k}^{2}}{2 \zeta _{\mathrm {k}}}-\sum _{k=1}^{n-1} \chi _{k}^{2}\left[ \frac{1}{\tau _{k}}-\frac{K_{e k}}{4}-\frac{A_{k}^{2}}{2 \iota _{k}^{2}}\right] \\&+\sum _{k=1}^{n-1} \xi _{k}+e_{n} K_{e n}\left[ \frac{{\kappa }_{n} e_{n} K_{e n} \theta _{n}^{*}}{2 \omega _{n}^{2} \varphi _{n}^{T}\left( \bar{x}_{n}\right) \varphi _{n}\left( \bar{x}_{n}\right) }+\frac{{\kappa }_{n} e_{n} K_{e n}}{2 \eta _{n}^{2}}+\frac{e_{n} K_{e n}}{2}+\frac{\chi _{n-1}}{\tau _{n-1}}+u\right. \\&\left. +\left( 1-q\left( e_{n}\right) \right) \frac{\dot{k}_{a n}(t)}{k_{a n}(t)} e_{n}+\left( q\left( e_{n}\right) \right) \frac{\dot{k}_{b n}(t)}{k_{b n}(t)} e_{n}\right] +\frac{\omega _{n}^{2}}{2}+\frac{\eta _{n}^{2} \bar{\lambda }_{n}^{2}}{2 {\kappa }_{n}}+\frac{\bar{\varepsilon }_{n}^{2}}{2}-\frac{\tilde{\theta }_{n} \dot{\theta }_{n}}{\zeta _{n}} \end{aligned}$$

The actual controller *u* and adaptive law $\dot{\theta }_{i}$ of the design system are as follows

62 $$\begin{aligned} u=&-\left( \sigma _{n}+v_{n}(t)\right) e_{n}-\frac{{\kappa }_{n} e_{n} K_{e n} \theta _{n}}{2 \omega _{n}^{2} \varphi _{n}^{T}\left( \bar{x}_{n}\right) \varphi _{i}\left( \bar{x}_{n}\right) } \nonumber \\&-\frac{{\kappa }_{n} e_{n} K_{e n}}{2 \eta _{n}^{2}}-\frac{e_{n} K_{e n}}{2}-\frac{\chi _{n-1}}{\tau _{n-1}}-\frac{K_{e n-1}}{K_{e n}} e_{n-1} \end{aligned}$$

63 $$ \dot{\theta }_{n}=\frac{\zeta _{n} {\kappa }_{n} e_{n}^{2} K_{e n}^{2}}{2 \omega _{n}^{2} \psi _{n}^{T}\left( \bar{x}_{n}\right) \varphi _{n}\left( \bar{x}_{n}\right) }-\gamma _{n} \theta _{n} $$

where $\sigma _{1}>0$ and $y_{1}>0$ design parameters, and the time-varying gain $v_{n}(t)$ is given by

64 $$ v_{n}(t)=\sqrt{\left( 1-q\left( e_{n}\right) \right) \left( \frac{\dot{k}_{a n}}{k_{a n}}\right) ^{2}+q\left( e_{n}\right) \left( \frac{\dot{k}_{b n}}{k_{b n}}\right) ^{2}+\zeta } $$

Substituting (62) and (63) into (61), we can obtain that

65 $$ \begin{aligned} \dot{V}_{n} \le&-\sum _{k=1}^{n-1}\left( \sigma _{k}-1\right) e_{k}^{2} {K}_{e k}-\sigma _{n} e_{n}^{2} {K}_{e n}\\&-\sum _{k=1}^{n-1} \frac{{\gamma }_{k} \tilde{\theta }_{k}^{2}}{2 \zeta _{k}}-\sum _{k=1}^{n-1} {\chi }_{k}^{2}\left[ \frac{1}{\tau _{k}}-\frac{{K}_{e k}}{4}-\frac{{A}_{k}^{2}}{2 {\iota }_{k}^{2}}\right] \\&+\sum _{k=1}^{n-1} \xi _{k}+\frac{\omega _{n}^{2}}{2}+\frac{\eta _{n}^{2} \bar{\lambda }_{n}^{2}}{2 {K}_{n}}+\frac{\overline{{\varepsilon }}_{n}^{2}}{2}+\frac{\gamma _{n} \theta _{n} \bar{\theta }_{n}}{\zeta _{n}}+\frac{{\iota }_{n}^{2}}{2} \end{aligned}$$

where

$$ \frac{\gamma _{n} \tilde{\theta }_{n} \theta _{n}}{\zeta _{n}}=\frac{\gamma _{n} \tilde{\theta }_{n}\left( \theta _{n}^{*}-\tilde{\theta }_{n}\right) }{\zeta _{n}} \le \frac{\gamma _{n} \theta _{n}^{* 2}}{2 \zeta _{n}}-\frac{\gamma _{n} \tilde{\theta }_{n}^{2}}{2 \zeta _{n}} $$

The updated (65) is designed as

66 $$\begin{aligned}  \dot{V}_{n} \le&- \sum _{k=1}^{n-1}\left( \sigma _{k}-1\right) e_{k}^{2} K_{e k}-\sigma _{n} e_{n}^{2} K_{e n}-\sum _{k=1}^{n} \frac{\gamma _{k} \tilde{\theta }_{k}^{2}}{2 \zeta _{k}} \\&-\sum _{k=1}^{n-1} \chi _{k}^{2}\left[ \frac{1}{\tau _{k}}-\frac{K_{e k}}{4}-\frac{A_{k}^{2}}{2 \iota _{k}^{2}}\right] +\sum _{k=1}^{n} \xi _{k} \end{aligned}$$

where

$$ \xi _{n}=\frac{\bar{\varepsilon }_{n}^{2}}{2}+\frac{\omega _{n}^{2}}{2}+\frac{\eta _{n}^{2} \bar{\lambda }_{n}^{2}}{2 \kappa _{n}}+\frac{Y_{n} \theta _{n}^{* 2}}{2 \zeta _{n}}+\frac{\iota _{n}^{2}}{2} $$

### Remark 3

In order to apply backstepping method to the design of controller for non-strict feedback nonlinear system, the control method proposed removes the limitation of the unknown functions $\left| f_{i}(x)\right| \le {\Phi }(|x|)$ in references^18,19^, which makes the proposed control scheme more widely used.

### Remark 4

Note that $\zeta$ is a positive constant and can guarantee $v_{1}(t)>0$ when $\dot{k}_{a l}$ and $\dot{k}_{b 1}$ are both zero.

### Remark 5

Note that $\zeta$ is a positive constant and can guarantee $v_{i}(t)>0$ when $\dot{k}_{a i}$ and $\dot{k}_{b i}$ are both zero.

## Stability analysis

### Theorem 1

For the non-strict feedback nonlinear system (1) with full state time-varying asymmetric constraints, under assumptions 2-3, according to the proposed control scheme, the actual controller (62), virtual control functions (24) and (46), adaptive laws (25), (47) and (63) can be designed to satisfy the control objectives.

### Proof

In order to facilitate the calculation process, the following parameters are defined.

67 $$ \bar{\sigma}_{i}=\sigma_{i}-1, i=1, 2, \cdots , n-1 $$

68 $$ \bar{o}_{n}=\sigma _{n} $$

69 $$ \bar{o}_{i}=\frac{1}{\tau _{i}}-\frac{K_{e i}}{4}-\frac{A_{i}^{2}}{2 \iota _{i}^{2}}, i=1,2, \cdots , n-1 $$

Then (66) can be expressed as follows

70 $$ \dot{V}_{n} \le -\delta V_{n}+\bar{\xi } $$

where $$\delta =\min \left\{ 2 \bar{\sigma }_{i}, \gamma _{i}(i=1,2, \cdots , n), 2 \bar{o}_{i}(i=1,2, \cdots , n-1)\right\} $$, $$\xi =\sum _{k=1}^{n} \xi _{k}$$

Then (70) can be obtained by integrating on [0, *t*]

71 $$ 0 \le V_{n}(t) \le \left( V_{n}(0)-\frac{{\bar{\xi }}}{\delta }\right) e^{-\delta t}+\frac{{\bar{\xi }}}{\delta },\forall {t} \ge \mathbf {0} $$

Based on lemma 3 and lemma 4, formula (70) and (71), this means that the variables $x_{i}$ , ${\theta }_{i}$ , ${\chi _{i}}$, $e_{i}$ and *u* are bounded. It can be further obtained

72 $$ \frac{1-q\left( e_{i}\right) }{2} \log \left( \frac{{k}_{a i}^{2}}{{k}_{a i}^{2}-{e}_{i}^{2}}\right) +\frac{q\left( e_{i}\right) }{2} \log \left( \frac{k_{b i}^{2}}{k_{b i}^{2}-z_{i}^{2}}\right) \le e^{2\left[ \left( V_{n}(0)-\frac{\bar{\xi }}{\delta }\right) e^{-\delta f}+\frac{\bar{\xi }}{\delta }\right] } $$

From (74), the tracking error ${e}_{i}$ satisfies

73 $$ \left| {e}_{i}({t})\right| \le {k}_{b i} \sqrt{1-{e}^{-2\left[ \left( V_{n}(0)-\frac{\bar{\xi }}{\varepsilon }\right) e^{-\delta t}+\frac{\bar{\xi }}{\delta }\right] }} $$

Because of $x_{1}(t)=e_{1}(t)+y_{r}(t)$, $z_{i}(t) \in Z_{i}=\left\{ -k_{a i}(t)<z_{i}<k_{b i}(t)\right\}$, $i=1,2, \cdots , n$ and according to Assumptions 2 and 3, we can obtain

74 $$ {k}_{c 1}(t) \le -{k}_{a 1}(t)+y_{r}(t)<x_{1}(t)<{k}_{b 1}(t)+y_{r}(t) \le \overline{{k}}_{c 1}(t),\forall {t} \ge \mathbf {0} $$

In the derivation process, it has been proved that $\alpha _{i}$, $i=1,2, \cdots , n$ is bounded, so it can be obtained that all states in the system (1) are satisfied

75 $$ \underline{k}_{c i}(t)<t_{i}(t)<\bar{k}_{c i}(t),\forall {t} \ge \mathbf {0} $$

$$\square $$

### Remark 6

It can be seen from (73) that the selection of upper and lower boundaries $k_{a i}$ and $k_{b i}$ of time-varying asymmetric constraint intervals will affect the tracking error of the system. According to Lemma 4 and (62) and the simulation results, when the constraint interval increases, the system tracking error increases and the system control effect becomes worse. When the constraint interval is reduced, the tracking effect of the system becomes better, but the peak and fluctuation of the system input *u* will become larger. Therefore, we should choose the appropriate constraint interval to balance the system.

## Simulation analysis

In this section, two simulation examples are given to demonstrate the effectiveness of the adaptive fuzzy controller proposed in this paper. Two control methods are adopted for each simulation example, and the two control methods are compared in the simulation results.

Case 1: The full state time-varying asymmetric constraint control scheme for non-strict feedback nonlinear systems based on the DSC proposed in this paper is applied.

Case 2: The traditional time-varying asymmetric constraint control scheme is used to the control of non-strict feedback nonlinear systems.

### Example 1: Numerical example

Consider the following non-strict feedback nonlinear state constrained system with external disturbances

76 $$ \left\{ \begin{array}{l} \dot{x}_{1}=x_{1} x_{2}^{2} \cos \left( x_{2}\right) +x_{2}+\varepsilon _{1} \\ \dot{x}_{2}=-x_{2} \sin \left( x_{2} x_{3}\right) +x_{3}+\varepsilon _{2} \\ \dot{x}_{3}=0.5 x_{1}+e^{x_{2} x_{3}}+u+\varepsilon _{3} \\ y=x_{1} \end{array}\right. $$

where $x_{1}$, $x_{2}$ and $x_{3}$ represent the state variables, *u* and *y* are the input and output of the system, respectively$\varepsilon _{1}=0.2 x_{1} \sin \left( x_{2}\right)$, $\varepsilon _{2}=0.1 x_{2} x_{3}$ and $\varepsilon _{3}=0.1 \cos \left( x_{2} x_{3}\right)$ are external disturbances, and the reference signal is $y_{r}=0.5 \cos (t)$.

The fuzzy membership functions are given as follows

77 $$ \mu _{F_{i}^{j}}\left( {x}_{i}\right) =\exp \left[ \frac{-\left( {x}_{i}+12-3 j\right) }{2}\right] ,j=1,2, \cdots ,7,i=1,2,3 $$

The virtual control functions $\alpha _{1}$, $\alpha _{2}$ actual controller *u* adaptive law $\theta _{1}$, $\theta _{2}$, $\theta _{3}$ of the system (76) are designed, and the design parameters are chosen as $\omega _{1}=3$, $\omega _{2}=2$, $\omega _{3}=2$, $\sigma _{1}=17$, $\sigma _{2}=10$,$\sigma _{3}=9$, $\eta _{1}=6$, $\eta _{2}=5$, $\eta _{3}=3$, $\zeta _{1}=0.5$, $\zeta _{2}=0.6$, $\zeta _{3}=0.6$, $\gamma _{1}=5$, $\gamma _{2}=3$, $\gamma _{3}=5$, $\tau _{1}=0.2$, $\tau _{2}=0.02$, $\zeta =10$.

The lower and upper bounds of the time-varying asymmetric constraint interval of the system are set as $\bar{k}_{c 1}=0.7+0.3 \cos (t)$, $\underline{k}_{c 1}=-0.6+0.2 \cos (t)$, $\bar{k}_{c 2}=0.8-0.3 \sin (t)$, $\underline{k}_{c 2}=0.7-0.5 \sin (t)$, $\bar{k}_{c 3}=1.5+1.2 \sin (t+0.5)$, $\underline{k}_{c 3}=-2+ \sin (t+5)$ respectively and the initial conditions are ${x}_{1}(0)=0.5$, ${x}_{2}(0)=0.5$, ${x}_{3}(0)=0$, $\theta _{1}(0)=0.01$, $\theta _{2}(0)=0.01$, $\theta _{3}(0)=0.01$.

**Figure 1:**
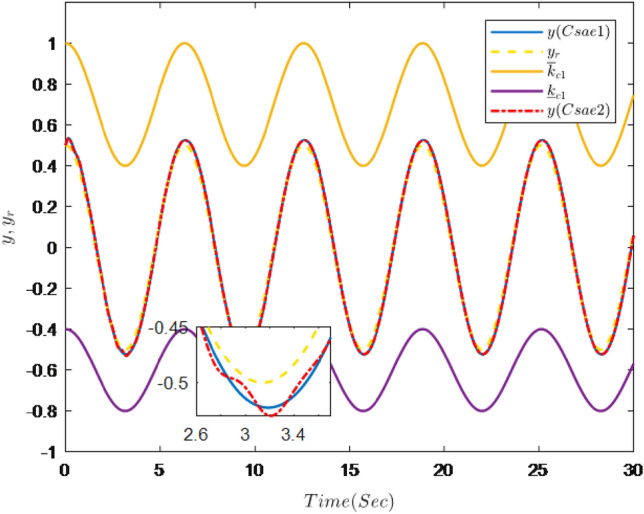
Trajectories of the output *y*, the reference signal $y_{r}$ and constraint interval.

**Figure 2:**
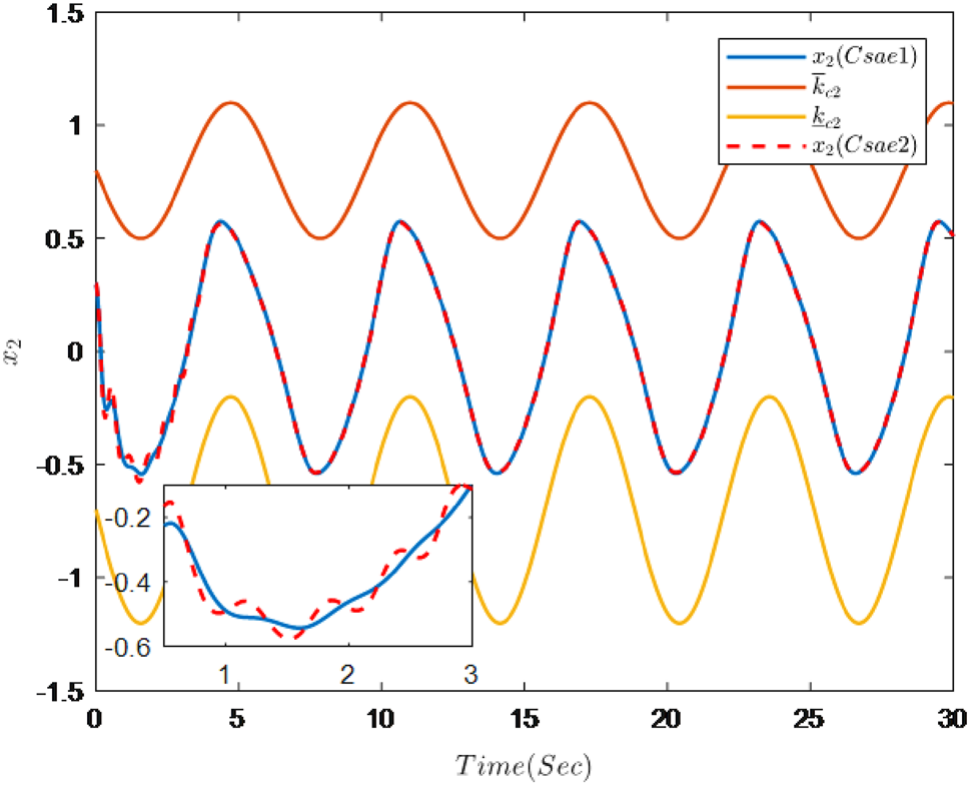
Trajectories of state $x_{2}$ and constraint interval.

**Figure 3:**
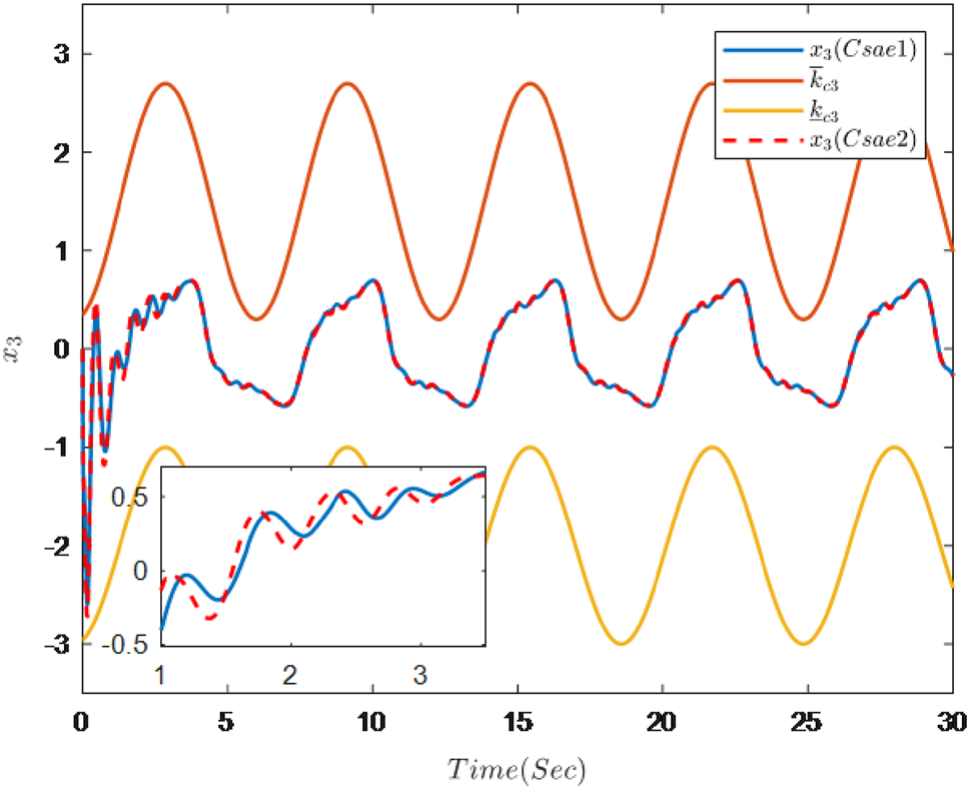
Trajectories of state $x_{3}$ and constraint interval.

**Figure 4:**
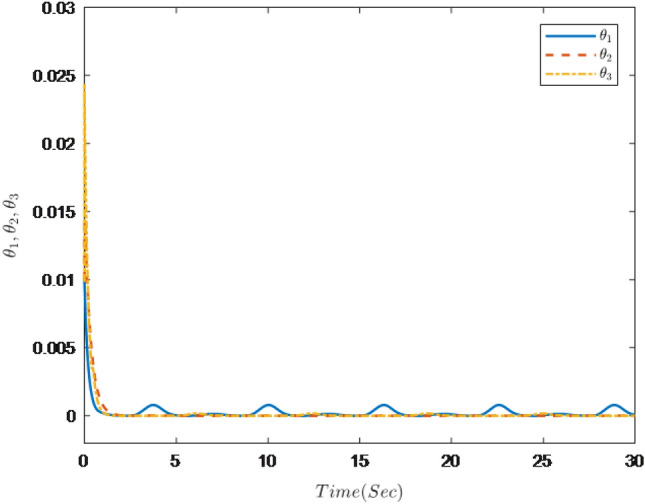
Trajectories of adaptive law $\theta _{1}$ $\theta _{2}$ $\theta _{3}$.

**Figure 5:**
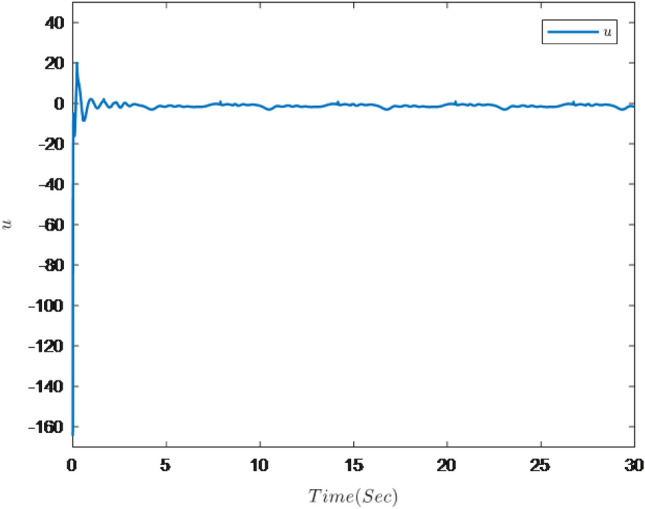
Trajectory of the system input *u*.

**Figure 6:**
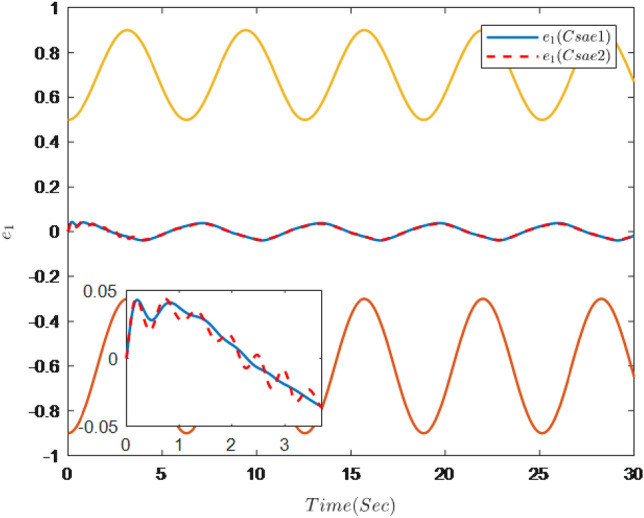
Trajectories of tracking error $e_{1}$ and constraint interval.

Figures 1, 2, 3, 4, 5 and 6 are the results of the simulation. Figure 1 shows the trajectories of the system output *y*, the reference $$y_{r}$$ and constraint intervals. Figures 2 and 3 are the trajectories of $$x_{2}$$ and $$ x_{3}$$ and constraint intervals. Figure 4 shows the trajectories of adaptive law $$ \theta _{1}$$, $$ \theta _{2}$$and $$ \theta _{3}$$. Figure 5 shows the trajectory of the system input *u*. Figure 6 shows the trajectory of tracking error $$e_{1}$$.

It can be seen from Figs. 1, 2, 3, 4, 5 and 6 that the controller designed in this paper can realize the effective tracking control of the non-strict feedback nonlinear system (76) with external disturbance. The system output can achieve the desired tracking effect, and the output tracking error do not violate constraint conditions. All variables of the system are bounded. Compared with the traditional time-varying asymmetric constraint control scheme, the time-varying asymmetric constraint control scheme based on DSC method can full states and the tracking error do not violate constraint conditions, and all variables of the system are bounded. The above numerical simulation shows that the adaptive fuzzy controller designed in this paper can satisfy the control requirements.

### Example 2: Application example

In the face of more and more complex production processes, the control requirements of industrial manipulators are also increasing. How to effectively control industrial manipulator has always been a hot research direction, and many research results have been obtained in recent years. In some work tasks that need to interact with people or high-precision, in order to ensure production safety and control accuracy, the motion space, motion speed and tracking error of the manipulator need to be limited. Therefore, it is of great practical significance to study the constraint control of manipulator.

Therefore, in the simulation design of this section, the system model of one-link manipulator^37–39^ is adopted, the adaptive fuzzy controller designed in this paper is applied to the control of one-link manipulator, and the time-varying asymmetric constraint interval is designed to restrict the rotation angle, rotation speed and torque of one-link manipulator.

The system model of one-link manipulator can be expressed as the following

78 $$ \left\{ \begin{array}{l} \dot{x}_{1}=x_{2} \\ \dot{x}_{2}=-10 \sin \left( x_{1}\right) -x_{2}+x_{1}^{2} \cos \left( x_{2} x_{3}\right) +x_{3} \\ \dot{x}_{3}=-2 x_{2}-10 x_{3}+10 u \\ y=x_{1} \end{array}\right. $$

where ${x}_{1}=q$ is the angular position of the one-link manipulator, ${x}_{1}=\dot{q}$ is the angular velocity, $x_{3}$ is the torque, and the reference signal is $y_{r}=0.5 \sin (t)$.

The fuzzy membership functions are given as follows

79 $$ \mu _{F_{i}^{\prime }}\left( x_{i}\right) =\exp \left[ \frac{-\left( x_{i}+3-j\right) }{2}\right] ,j=1,2, \cdots ,5,i=1,2,3 $$

The actual controller, virtual control function and adaptive laws of the one-link manipulator are designed according to the design method in this paper.

The design parameters are $\omega _{1}=2$, $\omega _{2}=6$, $\omega _{3}=3$, $\sigma _{1}=15$, $\sigma _{2}=10$, $\sigma _{3}=12$, $\eta _{1}=6$, $\eta _{2}=5$, $\eta _{3}=5$, $\zeta _{1}=0.1$, $\zeta _{2}=0.1$, $\zeta _{3}=0.2$, $\gamma _{1}=3$, $\gamma _{2}=1$, $\gamma _{3}=2$, $\tau _{1}=0.09$, $\tau _{2}=0.02$, $\zeta =5$. The initial conditions are ${x}_{1}(0)=0.01$, ${x}_{2}(0)=0.3$, ${x}_{3}(0)=0$, $\theta _{1}(0)=0.01$, $\theta _{2}(0)=0.01$, $\theta _{3}(0)=0.01$ the lower and upper bounds of the time-varying asymmetric constraint interval of the manipulator are $\bar{k}_{c 1}=0.5+0.2 \cos (t)$, $\underline{k}_{c 1}=-0.3+0.3\sin (t)$, $\bar{k}_{c 2}=0.5+0.5 \cos (t)$, $\underline{k}_{c 2}=-0.6+0.3 \cos (t)$, $\bar{k}_{c 3}=6+5 \sin (t)$, $\underline{k}_{c 3}=-5+ 3\sin (t)$.

**Figure 7:**
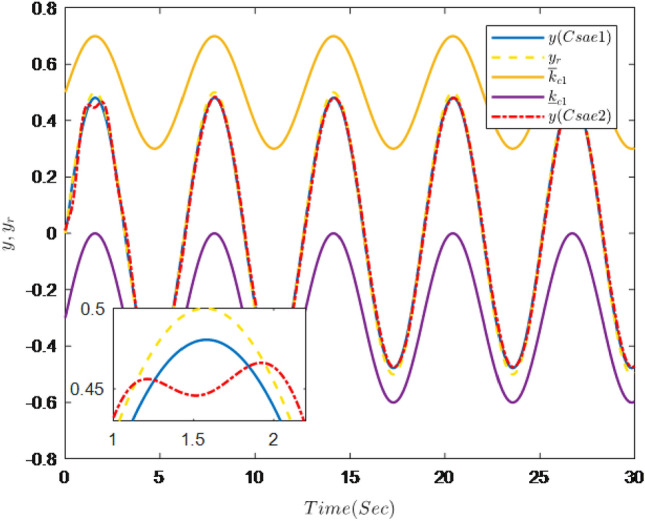
Trajectories of the output *y*, the reference signal $y_{r}$ and constraint interval.

**Figure 8:**
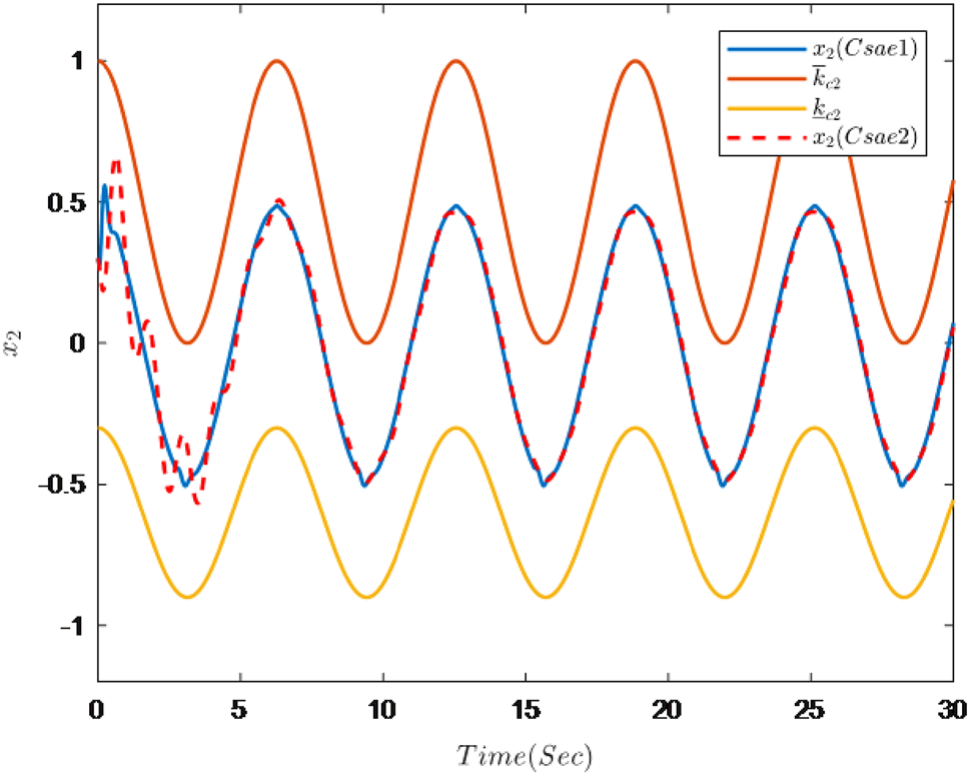
Trajectories of state $x_{2}$ and constraint interval.

**Figure 9:**
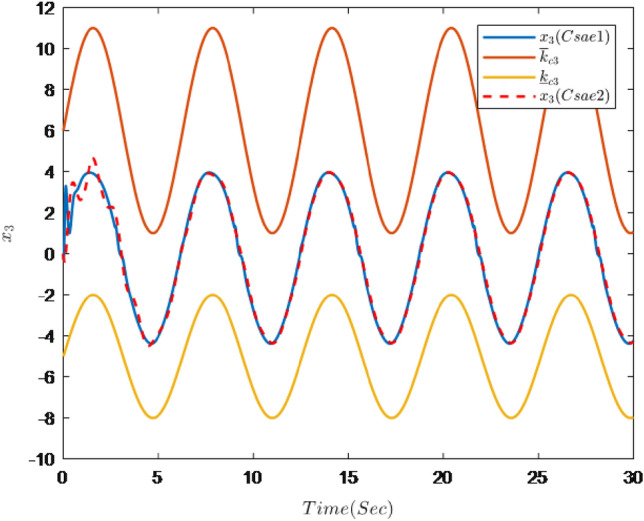
Trajectories of state $x_{3}$ and constraint interval.

**Figure 10:**
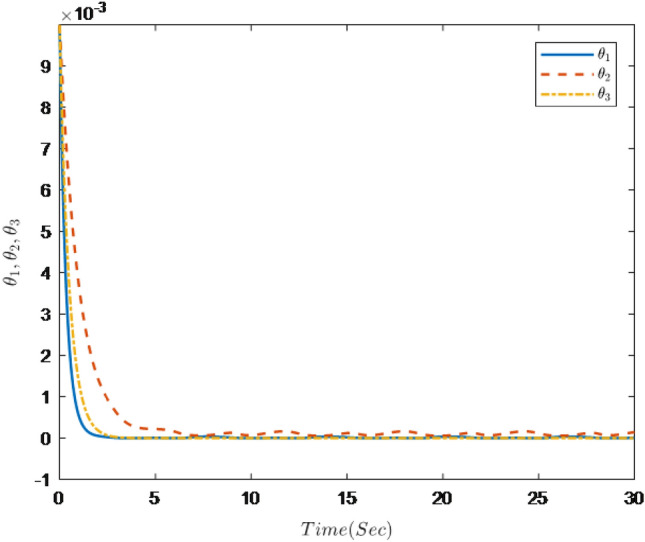
Trajectories of adaptive law $\theta _{1}$ $\theta _{2}$ $\theta _{3}$.

**Figure 11:**
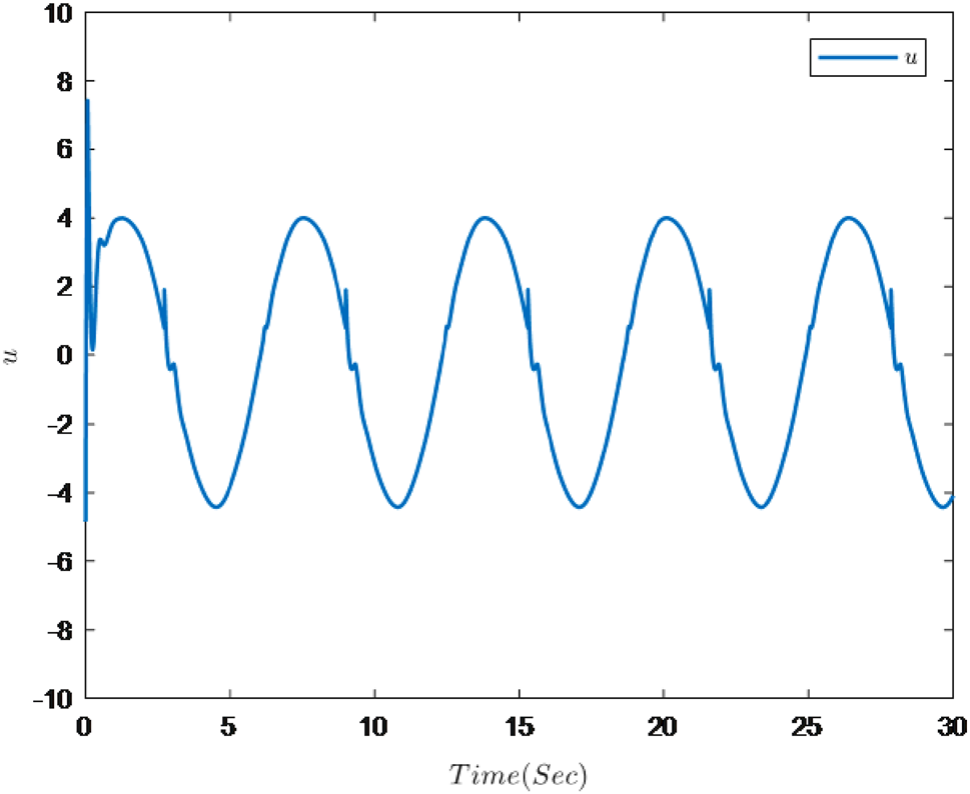
Trajectory of the system input *u*.

**Figure 12:**
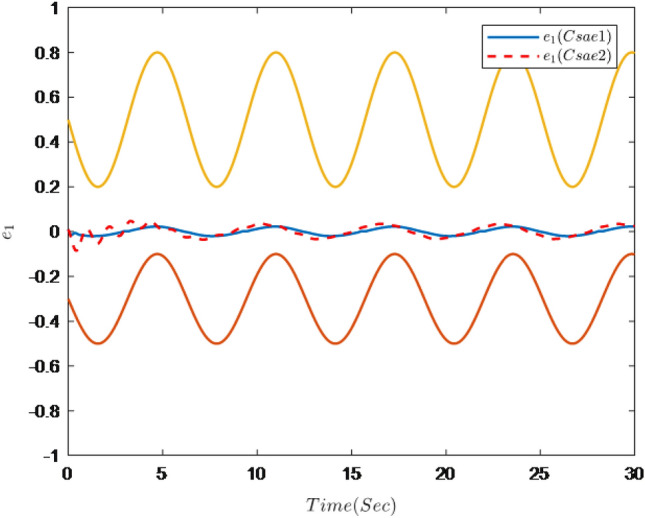
Trajectories of tracking error $e_{1}$ and constraint interval.

The simulation results are shown in Figs. 7, 8, 9, 10, 11 and 12.

Figure 7 shows the trajectories of the system output *y*, the reference $y_{r}$ and constraint interval.The adaptive fuzzy controller designed can ensure the one-link manipulator full state and the tracking error do not violate constraint conditions, and the system output $y_{r}$ can remain within a prescribed constraint interval. Figures 8 and 9 show are the trajectories of ${x}_{2}$ and ${x}_{3}$ and constraint intervals, system states ${x}_{2}$ and ${x}_{3}$ are constrained within intervals. Figures 10 and 11 shows the trajectories of adaptive law $\theta _{1}$, $\theta _{2}$ and $\theta _{3}$ and input *u*. It can be seen that all variables in the system are bounded. Figure 12 shows the trajectory of tracking error $e_{1}$, which satisfies the constraints. From the above simulation results, it can be seen that the time-varying asymmetric constraint control scheme based on the DSC method designed in this paper can effectively control the one-link manipulator, time-varying asymmetric constraints on the rotation angle, rotation speed and torque of the manipulator, and reduce the stabilization time of the one-link manipulator.

## Conclusion

In this paper, based on the DSC method, time-varying asymmetric constraints are applied to a class of non-strict feedback nonlinear systems. In the design process, the fuzzy logic system is used to estimate the unknown nonlinear function in the system. In each step of the controller design process, the time-varying asymmetric BLF is introduced to design the lower and upper time-varying constraint boundaries of the system state respectively, in order to time-varying asymmetric constraints on all states of the system. Based on the DSC method, a first-order filter is introduced to process the virtual control function in the design process, which solves the problem that the traditional adaptive backstepping design method needs to perform repeated differential calculations on the virtual control function, reduces the order of TABLF, reduces the computational burden and speeds up the tracking speed of the system. Finally, through numerical simulation and one-link manipulator system simulation, it is proved that the adaptive fuzzy controller designed in this paper can meet the predetermined control requirements. The simulation results show that all signals of the system are bounded, and all states of the system do not violate the time-varying asymmetric constraints during operation. The adaptive tracking control for a class of switch nonlinear systems or stochastic nonlinear system with full state constraints will be our future works.

## Data Availability

All data generated or analysed during this study are included in this published article.
